# Precursor Resolution via Ion Z‐State Manipulation: A Tandem Mass Spectrometry Approach for the Analysis of Mixtures of Multiply‐Charged Ions

**DOI:** 10.1002/jms.5124

**Published:** 2025-03-30

**Authors:** Nicolas J. Pizzala, Hsi‐Chun Chao, Boukar K. S. Faye, Scott A. McLuckey

**Affiliations:** ^1^ Department of Chemistry Purdue University West Lafayette Indiana USA; ^2^ Beckman Institute for Advanced Science and Technology University of Illinois Urbana‐Champaign Illinois USA

**Keywords:** charge reduction, electrospray ionization, mixture analysis, tandem mass spectrometry

## Abstract

Electrospray ionization (ESI) is often the ionization method of choice, particularly for high‐mass polar molecules and complexes. However, when analyzing mixtures of analytes, charge state ambiguities and overlap in mass‐to‐charge (*m/z*) can arise from species with different masses and charges. While solution‐phase conditions can sometimes be optimized to produce relatively low charge states—thereby reducing charge‐state ambiguity and *m/z* overlap—gas‐phase methods offer greater control over charge state reduction. For complex mixtures, however, charge state reduction alone often fails to resolve individual components in the mixture. Incorporating a mass‐selection step prior to charge state manipulation can simplify the mixture and significantly improve the separation of the components. This general tandem mass spectrometry approach is referred to here as **p**recursor **r**esolution via **i**on **z**‐state **m**anipulation (**PRIZM**). Examples of variations of PRIZM experiments date back roughly 25 years and have involved ion/molecule proton transfer reactions, ion/ion proton transfer reactions, ion/ion electron transfer reactions, electron capture reactions, and multiply‐charged ion attachment reactions. This tutorial review describes the PRIZM approach and provides illustrative examples using each of the charge state manipulation approaches mentioned above.

## 1 Introduction

Electrospray ionization (ESI) [1] is among the most impactful developments in mass spectrometry by virtue of the wide range of important applications that it has enabled. The ability to generate ions directly from solution provides means for producing ions from polar nonvolatile molecules that greatly facilitates the coupling of condensed‐phase separations, such as liquid chromatography [2, 3, 4] and capillary electrophoresis [5, 6], with mass spectrometry. ESI also provides a soft ionization approach for many classes of polymers [7], including biopolymers [8, 9], and complexes derived therefrom [10]. A hallmark of ESI is its tendency to generate multiply‐charged ions from molecules with multiple polar sights. The multiple‐charging phenomenon has several profound implications for mass spectrometry and tandem mass spectrometry. For example, multiple‐charging reduces mass‐to‐charge (*m/z*) ratios, which decreases the upper *m/z* requirement for the mass analyzer. Multiple‐charging allows for higher mass resolution measurements for analyzers that provide better resolution at low *m/z* (e.g., Fourier transform ion cyclotron resonance [FT‐ICR] [11] and electrostatic ion traps [12], including the Orbitrap™ [ 13], mass analyzers) and improves detection efficiency both for approaches based on image current measurements, such as the FT‐ICR and Orbitrap, as well as for approaches that rely on electron multiplication [14]. The multiple‐charging phenomenon also affects the kinetic stability of the molecule‐ion and influences favored dissociation channels. As a result, tandem mass spectra (MS/MS spectra) and the structural information derived therefrom are charge‐state dependent [15]. The consequences of multiple‐charging listed above generally facilitate the determination of the masses and primary structures of analyte ions. However, the multiple‐charging phenomenon introduces ambiguity in determining mass from the measurement of *m/z* due to the fact that both mass and charge are, a priori, unknown. For this reason, charge state determination is an essential element in many applications of ESI mass spectrometry and tandem mass spectrometry. In this review, approaches to determining charge states in ESI‐MS and ESI‐MS/MS are briefly reviewed, and a type of MS/MS experiment, referred to herein as precursor resolution via ion Z‐state manipulation (PRIZM), is described that facilitates analyte mass determination of multiply‐charged analytes in mass spectra that otherwise are too convoluted to make mass measurements.

### 1.1 Charge State Determination in ESI‐MS and ESI‐MS/MS

**FIGURE 1:**
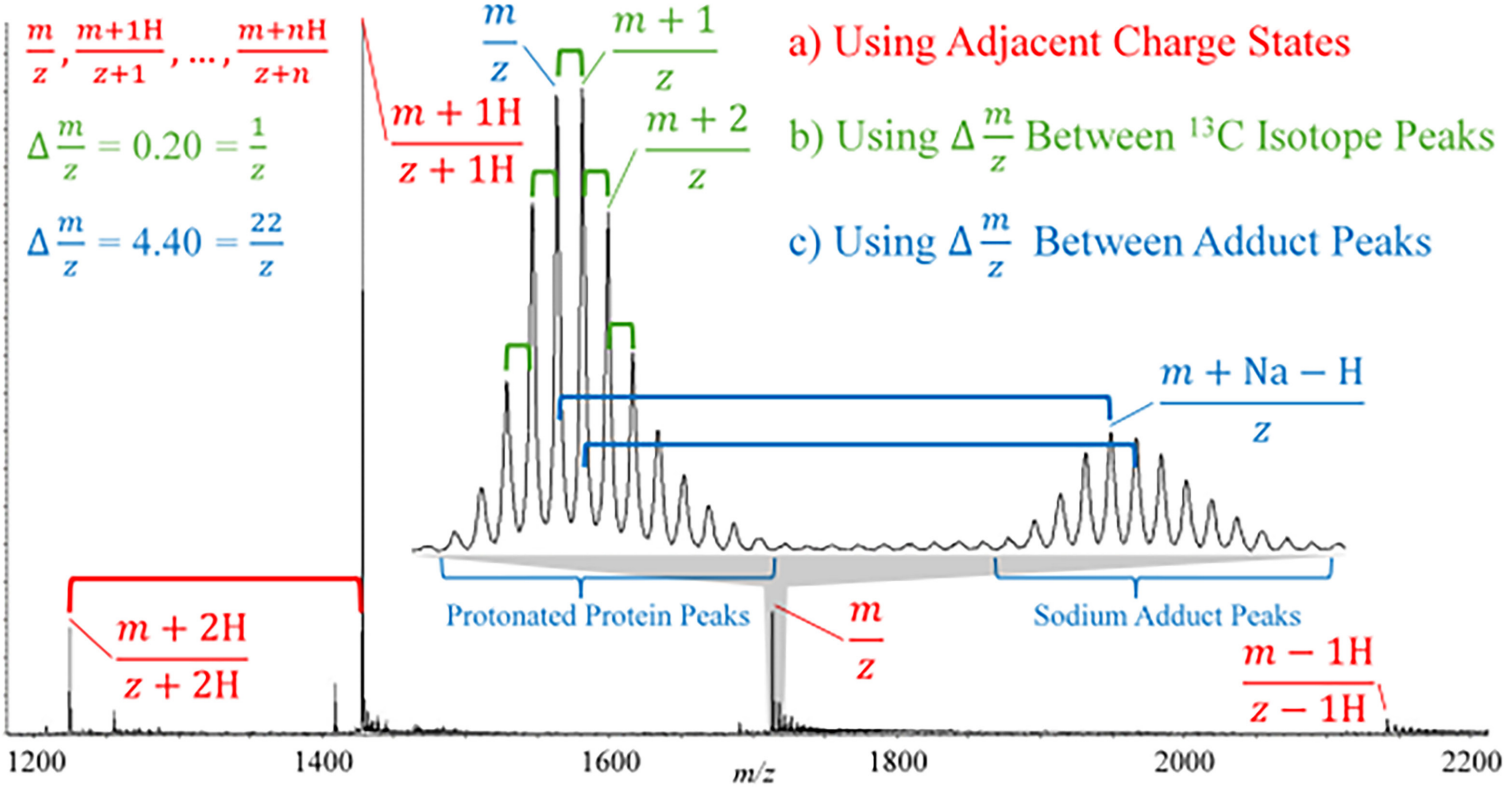
ESI/MS illustrating the three common means for determining charge states from peak spacings (i.e., [a] spacing between adjacent charge states, [b] spacings between isotopes, and [c] spacing between adduct peaks).

A variety of approaches have been developed to determine charge states of ions in mass spectra generated via ESI. It was shown early in the application of ESI to proteins that the presence of two or more ions from the same molecule with known differences in mass and charge allows for the determination of ion charge via the solution of a set of simultaneous equations [9, 16]. A variety of algorithms have since been described that can be used to automatically determine the mass of an analyte from the most probable charge state distribution based on Bayesian statistics [17, 18, 19, 20]. The output of such algorithms is commonly referred to as a “zero‐charge deconvolution”. An analogous approach takes advantage of the presence of an adduct ion within a specific charge state, characterized by a known mass difference (e.g., the replacement of a proton with a sodium ion leading to a mass difference of 22 Da). The *m/z* spacing between the adducted and nonadducted ion reveals the charge state. Moreover, even when an adduct of known mass is not present, the *m/z* spacings between adjacent isotope peaks can reveal the ion's charge [21]. ESI mass spectra were first collected using low resolution mass analyzers, which generally precluded charge determination via isotope spacings for ions greater in mass than a few kiloDaltons. The coupling of ESI with high‐resolution instruments has significantly extended the range of analyte masses for which isotope spacing measurements can be used to determine charge states [22]. Figure 1 provides an example of charge state determination via (a) *m/z* measurement of adjacent charge states, (b) *m/z* spacings between isotope peaks for a given charge state, and (c) via *m/z* spacings of adduct ions for a given charge state.

The preceding approaches rely on differences between *m/z* values determined from measurements of many ions (i.e., experiments involving ion ensembles) to determine charge and are most relevant to the subject of this tutorial review. An alternative approach relies on the independent measurement of ion *m/z* and *z* using image current detection. This approach is referred to as charge detection mass spectrometry (CDMS) [23, 24] and has been implemented with electrostatic linear ion traps as well as Orbitraps™ [25, 26]. This approach relies on the measurement of the charge of individual ions and is generally performed with only one [27] or a few ions [28] present in the ion trap at a time. This one‐at‐a‐time approach is particularly effective for relatively highly charged ions and has therefore been demonstrated to be especially advantageous for the mass measurement of large and heterogeneous species such as virus particles and nanoparticles [29]. Since CDMS is not an ion ensemble measurement, that is, hundreds or thousands of ions measured simultaneously, experiments involving complex heterogenous mixtures will tend to be long. Conversely, PRIZM is intended to extend the utility of classic mass spectrometry approaches to determine charge state information rather than utilizing new instrumentation platforms.

The classic tandem mass spectrometry (MS/MS) experiment, which involves the *m/z* selection of a precursor ion, activation, and dissociation of the precursor ion, and *m/z* analysis of the product ions, when applied to a multiply‐charged precursor ion, raise the challenge of determining the charges of the product ions. Provided the analyzer resolution is sufficient to resolve isotope spacings and there is minimal overlap between ions of different masses and charges (but still overlapping *m/z* ratios), product ion charge states can be determined from the magnitudes of the spacings between the isotope peaks. When the analyzer resolution is insufficient to resolve isotopes or the analyte mixture is so heterogeneous such that spectral overlap causes peaks to remain unresolved, product ion charge state determination becomes more challenging. While the charge state of the precursor ion places a constraint on the possible charge states of the product ions, distributions of product ion charge states with spacings of unit charge are usually absent, as are ions with adducts of known mass. Hence, two of the above approaches (Figure 1a,c) that can be used to determine charge states in mass spectra are generally unavailable in assigning charge states in product ion spectra. The first MS/MS platform coupled with ESI was the triple quadrupole, which was not capable of resolving isotope spacings for high‐mass product ions. Therefore, the initial applications of tandem mass spectrometry to multiply‐charged protein ions were restricted to generating “fingerprints” of precursor ions (i.e., no product ion mass assignment) [30]. It was not until ESI was coupled with the FT‐ICR platform that applications like top–down MS/MS of proteins could be pursued, since the higher resolution afforded the use of isotope spacings of product ions for charge state assignment [31].

### 1.2 Charge State Manipulation to Facilitate Charge State Determination in ESI‐MS(/MS)

As ESI‐MS(/MS) applications have evolved, a number of approaches intended to manipulate charge states have been developed to facilitate charge state assignment using one or more of the *m/z* spacing strategies described above. Among these approaches in ESI‐MS is the adjustment of solution conditions, often changes to pH, to lead to lower charge states, which are more readily resolved [32, 33, 34, 35, 36, 37]. The extent to which charge states can be reduced via altering ESI solution conditions alone, however, is relatively limited. Charge state distributions have been altered by exposing electrospray droplets to acidic [38], basic [39], or organic [40] vapors. In such cases, both droplet chemistry and gas‐phase ion/molecule chemistry can affect the charge states. For example, changes in droplet pH can result in changes in protein conformational states in the droplets [41], which can play a role in the mechanism by which ions emerge from the droplets [42]. The reduction of charge states generated via ESI has also been achieved using either ion/molecule proton transfer reactions [43, 44] or ion/ion proton transfer reactions [45, 46, 47, 48, 49, 50, 51] prior to admitting ions into the mass spectrometer.

Charge reduction strategies have also been implemented within mass spectrometers, which has the advantage of separating the charge reduction step from the processes associated with initial ion formation, desolvation, and transmission into the mass spectrometer. For product ion charge determination in tandem MS, the charge reduction step must take place within the mass spectrometer. The first examples of charge reduction within a mass spectrometer involved ion/molecule proton transfer reactions [52, 53, 54], including proton transfer for determining product ion charge states in tandem mass spectrometry [55]. Ion/molecule reactions within a mass spectrometer are usually implemented in instruments with the ability to trap ions, which allows for control of the reaction time along with the number density of the neutral reagent. Ion/molecule reactions, however, can be limited in the extent to which charge can be reduced and ion/molecule attachment can compete with proton transfer, which can lead to ambiguities in interpretation [56, 57]. Ion/ion proton transfer reactions, on the other hand, can reduce charge states to arbitrarily low values, although ion attachment can compete with proton transfer, depending upon the nature of the reagent ion [58].

Ion/ion proton transfer reactions in electrodynamic ion traps have been used extensively for the manipulation of charge states of multiply‐charged ions [59, 60, 61], including product ions generated in a tandem mass spectrometry experiment [62, 63, 64, 65, 66]. The reactions can take place under mutual ion polarity storage conditions, which can be established in 3‐D quadrupole ion traps and linear quadrupole ion traps with radio‐frequency voltages applied to the end‐plates [67] or in “transmission mode” where the analyte ions are stored in the ion trap while the reagent ions are transmitted through the ion trap such that there is overlap between the analyte and reagent ions [68, 69]. The electrodynamic ion trap environment also allows for the inhibition of ion/ion reactions on the basis of mass‐to‐charge thereby enabling so‐called “ion parking” experiments [70, 71, 72, 73, 74], which provides additional flexibility in multistage MS experiments [75]. Proton transfer, either via ion/molecule or ion/ion reaction, involves a change in charge (Δ*z*) of ± 1, depending upon the analyte ion polarity, and a mass change (Δ*m*) of ± 1 Da (i.e., the mass of a proton), depending upon whether the proton is lost or gained by the analyte ion. Electron‐based reactions, either via electron capture by a cationic analyte (Δ*m* = +5.486x10^−4^ Da; Δ*z* = −1) or ion/ion electron transfer (Δm ± 5.486x10^−4^ Da; Δ*z* ± 1, depending upon the analyte charge), can also achieve charge reduction. However, electron capture and electron transfer reactions involving multiply‐charged analyte ions tend to result in analyte ion fragmentation (viz. electron capture dissociation [76, 77] [ECD] and electron transfer dissociation [78, 79] [ETD]). As the size of the analyte ion increases, however, both electron capture and electron transfer without obvious dissociation (ECnoD and ETnoD, respectively) effectively result in charge reduction. Particularly for large analyte ions of low relative charge, as are often encountered in native mass spectrometry [80, 81, 82], ECnoD [83, 84] and ETnoD tend to dominate over ECD and ETD, respectively. The attachment of oppositely charged ions [85] (Δ*m* = mass of the reagent ion, Δ*z* = charge of the reagent ion) can also be used to reduce precursor ion charges. Ion attachment and proton transfer, for example, can be competitive processes, depending upon the sizes and the acidities/basicities of the charge bearing sites [86]. However, particularly for large analyte ions of low relative charge, ion attachment tends to dominate [87, 88].

## 2 Precursor Resolution via Ion Z‐State Manipulation (PRIZM)

The PRIZM experiment described here facilitates mass measurements in scenarios in which the ESI‐MS yields a broad unresolved signal, often referred to as a “blob” that precludes mass determination of many or all components that may be present. This scenario can arise from highly heterogeneous mixtures that, at the extremes, can be comprised of (i) a single, typically high mass, component that is itself highly heterogeneous or (ii) many individual components, such as a mixture of many small to moderate size polymers. The former case is often encountered in, for example, native MS applications, whereas the latter applies to polymer or protein mixture analysis. PRIZM involves the selection of a narrow range of *m/z* values followed by charge reduction. The *m/z* selection step simplifies the mixture of ions subjected to charge reduction, which facilitates the resolution of the charge states formed via charge reduction. There is an interplay between the width of the *m/z* selection and the extent of charge reduction. As the charge is reduced, the *m/z* spacing between charge states increases along with the *m/z* values of the ions. Provided the upper *m/z* range of the analyzer can accommodate the charge‐reduced ions and the detection method is capable of responding to the ions, the “peak capacity” of the measurement increases with the extent of charge reduction [89]. Hence, there is an inverse relationship between the complexity of the mixture of ions initially selected for reaction via the *m/z* selection step and the extent of charge state reduction.

### 2.1 PRIZM and a “Single” Heterogeneous Component

**FIGURE 2:**
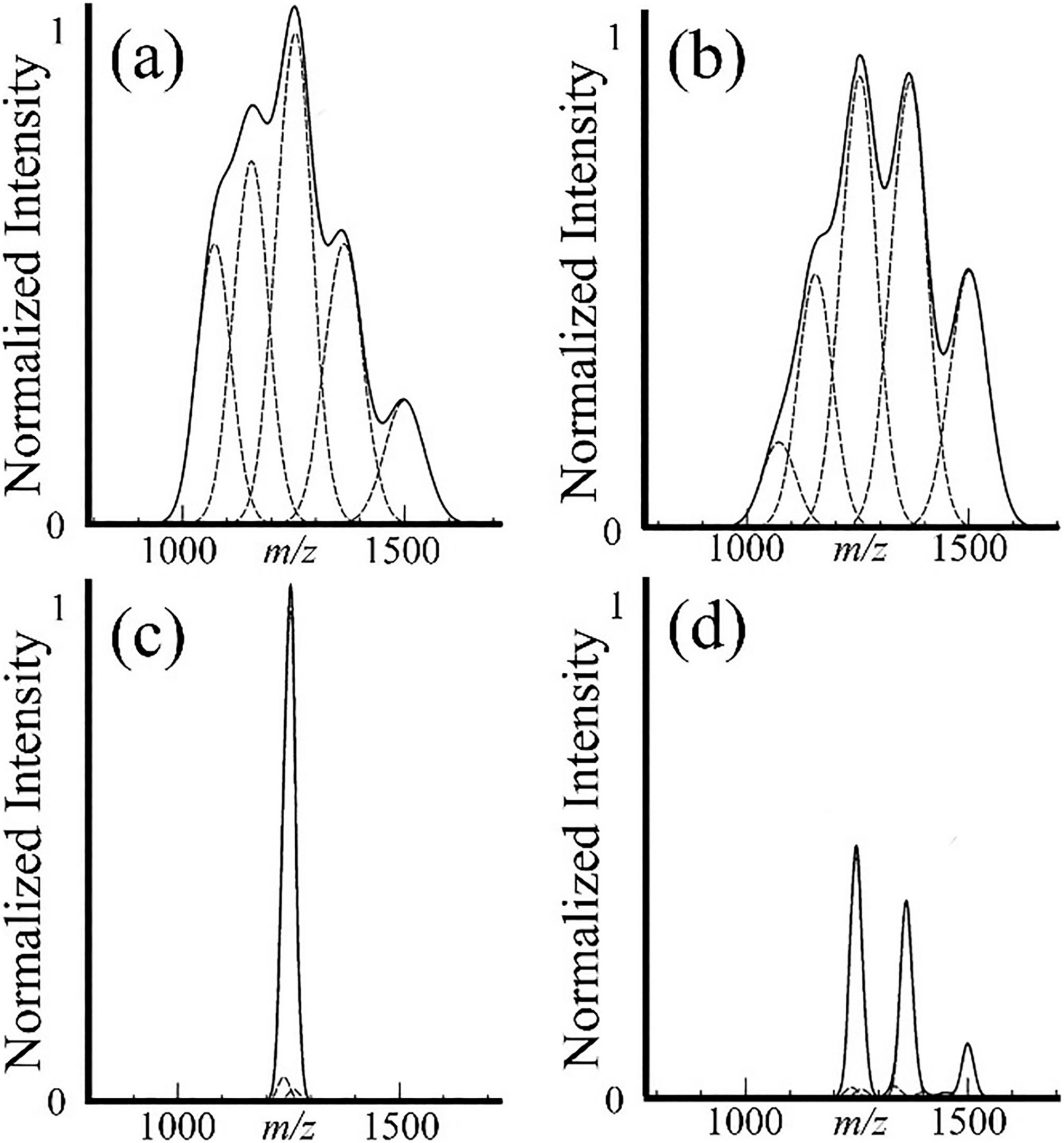
Illustration of a PRIZM experiment. (a) ESI‐MS of a roughly 15 kDa heterogeneous polymer with extensive charge state overlap. (b) ESI‐MS after brief ion‐molecule reaction time that shifts the abundance‐weighted average charge to a lower value. (c) Selection of a relatively narrow slide of *m/z* in the spectrum of (a). (d) ESI‐MS after the same ion/molecule reaction conditions of (b) for the ions in (c). Adapted with permission from Reference 90, copyright American Chemical Society.

The first description of a PRIZM experiment, although the term was not used to describe it, involved ion/molecule proton transfer as the means for charge state reduction [90]. Figure 2 is an adaptation of the original description of a PRIZM experiment based on a simulated ion/molecule proton transfer experiment with and without an initial mass‐selection step of a highly heterogeneous hypothetical analyte (see reference 90 for details). Figure 2a shows an initial ESI‐MS of an analyte that shows a range of Gaussian charge state peaks with unresolved isotopes that lead to significant charge state overlap. Figure 2b shows the charge state distribution after an ion/molecule proton transfer reaction period, which leads to a modest shift in the abundances of the charge states to lower charge. Extensive overlap between the charge states remains. Figure 2c shows the isolation of a narrow range of ions near the most intense region of Figure 2a. This step simplifies the mixture of ions that are subjected to charge state reduction. Figure 2d shows the spectrum resulting from the same proton transfer conditions used to generate Figure 2b. The product ions in Figure 2d show near baseline resolution, which leads to a more confident charge state assignment from the spacings of the adjacent charge states (see Figure 1).

**FIGURE 3:**
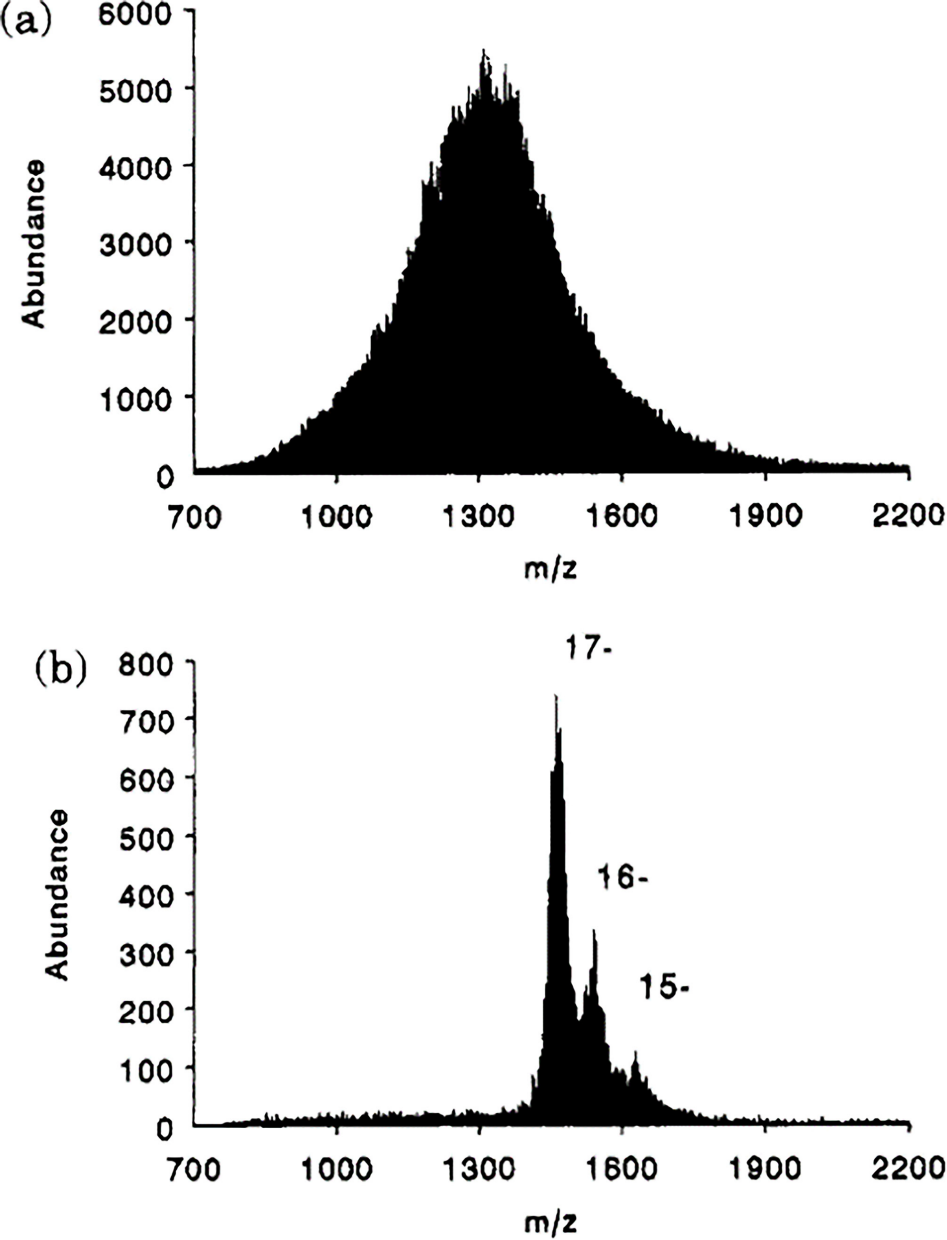
(a) Electrospray mass spectrum of the anions derived from *E. coli* tRNA, strain W. (b) Spectrum resulting from the mass selection of a narrow range of mass‐to‐charge values from the distribution of (a) followed by proton transfer reactions with trifluoro‐acetic acid. Adapted with permission from Reference 90, copyright American Chemical Society.

An experimental demonstration of PRIZM using ion/molecule proton transfer as the charge reduction mechanism was provided by anions generated from negative ESI of *Escherichia coli* (*E. coli*) tRNA strain W [90]. The ESI‐MS was generated under conditions that lead to a distribution of metal ion adduction, which resulted in extensive overlap between charge states and an unresolved blob in the spectrum (see Figure 3a). This constitutes an example of a “single” (i.e., tRNA) major component with a high degree of heterogeneity. After an ion isolation step and storage in an electrodynamic ion trap in the presence of trifluoro‐acetic acid, proton transfer from the neutral acid to the tRNA anions resulted in the spectrum of Figure 3b. Based on the spacings of the partially resolved product ions, charge state assignments could be made, which enabled the determination of mass.

**FIGURE 4:**
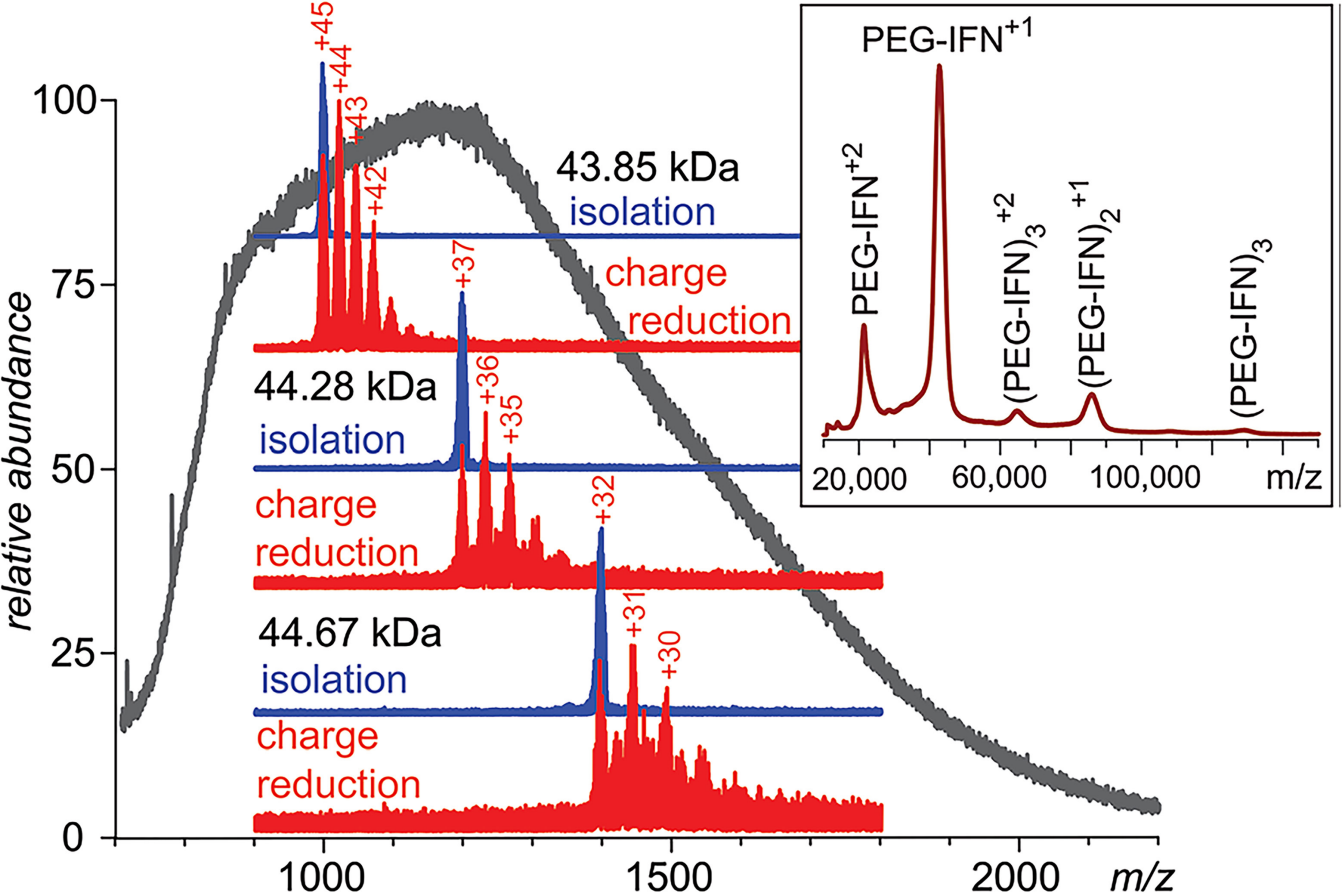
PEG‐IFN ESI‐MS (gray trace), ion isolations (blue traces), and respective post‐ion/ion reaction spectra (red‐traces). PEG‐IFN MALDI‐TOF‐MS (insert). Reproduced from Ref. 92 with permission from the Royal Society of Chemistry.

Ion/ion reactions are a more robust means for reducing charge states than are ion/molecule reactions for both thermodynamic and kinetic reasons (see reference 58). Kaltashov et al. demonstrated PRIZM experiments involving glycosylated [91] and PEGylated [92] proteins using limited charge reduction via ion/ion electron transfer as the means for charge reduction. Figure 4 summarizes a series of PRIZM experiments applied to a broad signal generated via ESI of PEGylated interferon β‐1a (PEG‐IFNβ). The gray trace shows the total ESI signal as a function of *m/z*, the blue traces show 10 *m/z* unit‐wide regions that were selected for charge reduction, and the red traces show the respective products following a limited ion/ion electron transfer period. The insert shows a MALDI‐TOF spectrum of the same sample. This figure illustrates the generation of mass information from the ESI of a large heterogeneous analyte that is not available from the “blob” (gray trace) generated directly from the sample. Notably, while the data from Figure 4 were itself collected via direct sample infusion, most of the data reported in the work from which Figure 4 was reproduced were collected during an on‐line chromatographic separation, which demonstrated PRIZM on the chromatographic time‐scale.

**FIGURE 5:**
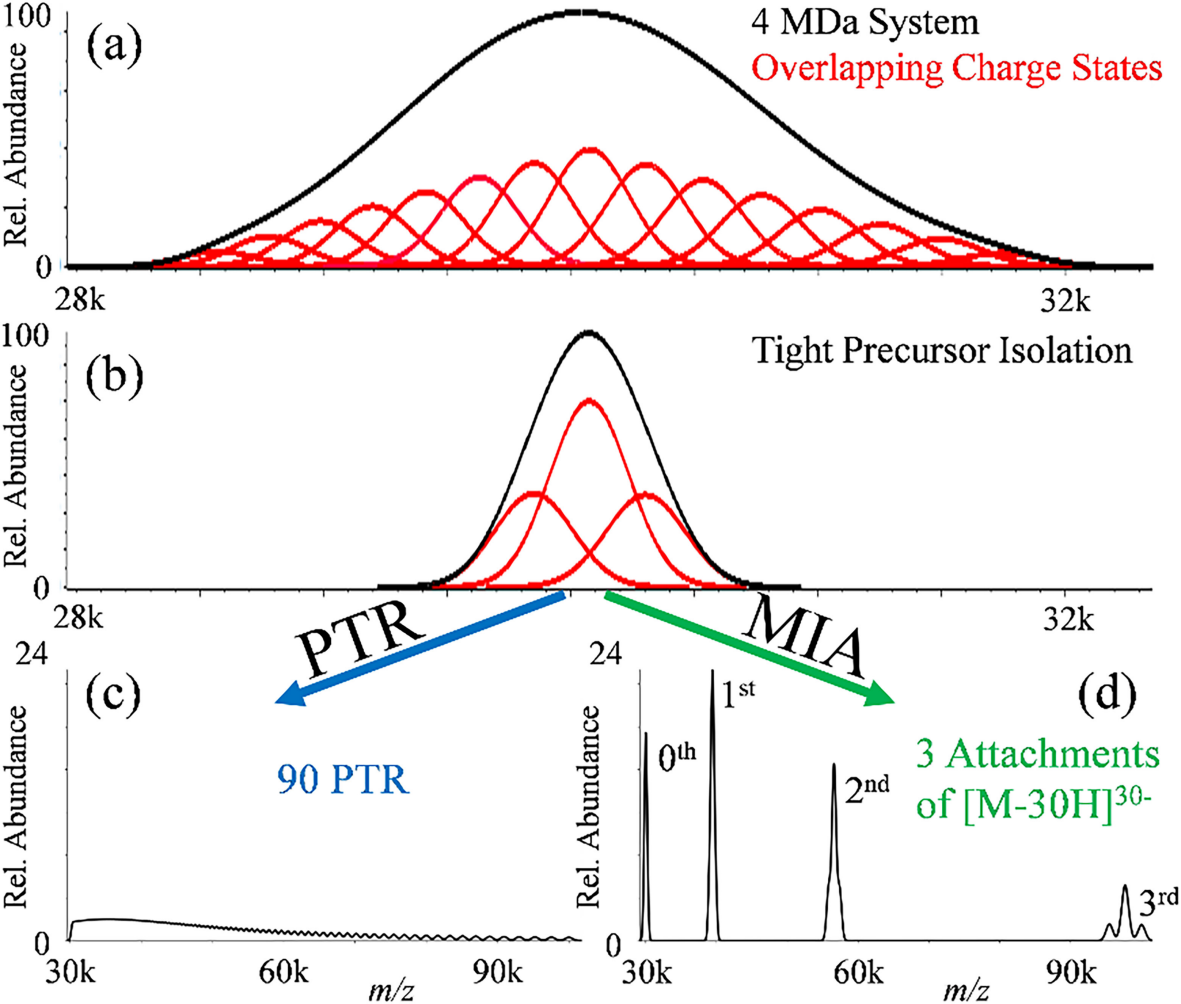
(a) Simulated ESI‐MS of a 4 MDa analyte (*z* = 126–140) with a FWHM of 50 kDa. (b) Selection of a *m/z* 1000 window from the mass spectrum. (c) Post‐ion/ion MS following up to 90 consecutive proton transfer reactions. (d) Post‐ion/ion MS following up to three attachments of a 30^−^ reagent anion of mass = 67 kDa.

Depending upon the size(s) and heterogeneity of the analytes, limited charge reduction through proton transfer or electron transfer/capture may not suffice to resolve charge states. In such cases, charge reduction might only lead to a slight shift of the unresolved “blob” to higher *m/z* without significantly improving charge state resolution. To address this, it may be advantageous to use larger step sizes in charge reduction, creating larger spacings between adjacent charge state peaks, and to minimize the range over which the analytical signal is dispersed. The attachment of multiply‐charged reagent ions of known mass and charge to analyte ions of opposite polarity allow for the generation of well‐defined changes in *m/z* that are much greater than those associated with the single‐charge‐at‐a‐time approaches discussed above. A PRIZM experiment using multiply‐charged ion attachment (MIA) as the charge reduction approach is contrasted with proton transfer experiment in the simulation provided in Figure 5. A 4‐MDa analyte species with a 50‐kDa FWHM with 126–140 charges is shown at the top (a). A 1000 *m/z* selection at the most abundant part of the top trace is shown directly below followed by up to 90 proton transfers (c) or MIA with up to three attachments of a 30^−^ reagent ion of roughly 67 000 Da (e.g., bovine serum albumin). The MIA approach, which provides flexibility in choosing the magnitudes of Δ*m* and Δ*z*, facilitates the resolution of charge states and allows for the determination of mass using the known values of reagent mass and charge [93]. This reaction is typically carried out by first generating analyte ions, isolating a narrow population either through a RF/DC mass filter or by ejecting unwanted ions through resonant excitation frequency sweeps [94] then cooling the selected population in a higher pressure (2–10 mTorr) ion trap; the oppositely charged reagent ions are then generated, isolated via the RF/DC mass filter tuned for the desired *m/z* of the reagent, then mutually stored in the same ion trap with the previously formed/isolated analyte ions. The application of an AC waveform, typically 100–300 kHz with 100–300 V_0‐p_, to orthogonal plates on either end the ion trap allow for the mutual storage of both positive and negative ions, providing trapping in the z‐direction [64, 67]. Mutual storage times can further be tuned to allow more or less reaction to occur.

**FIGURE 6:**
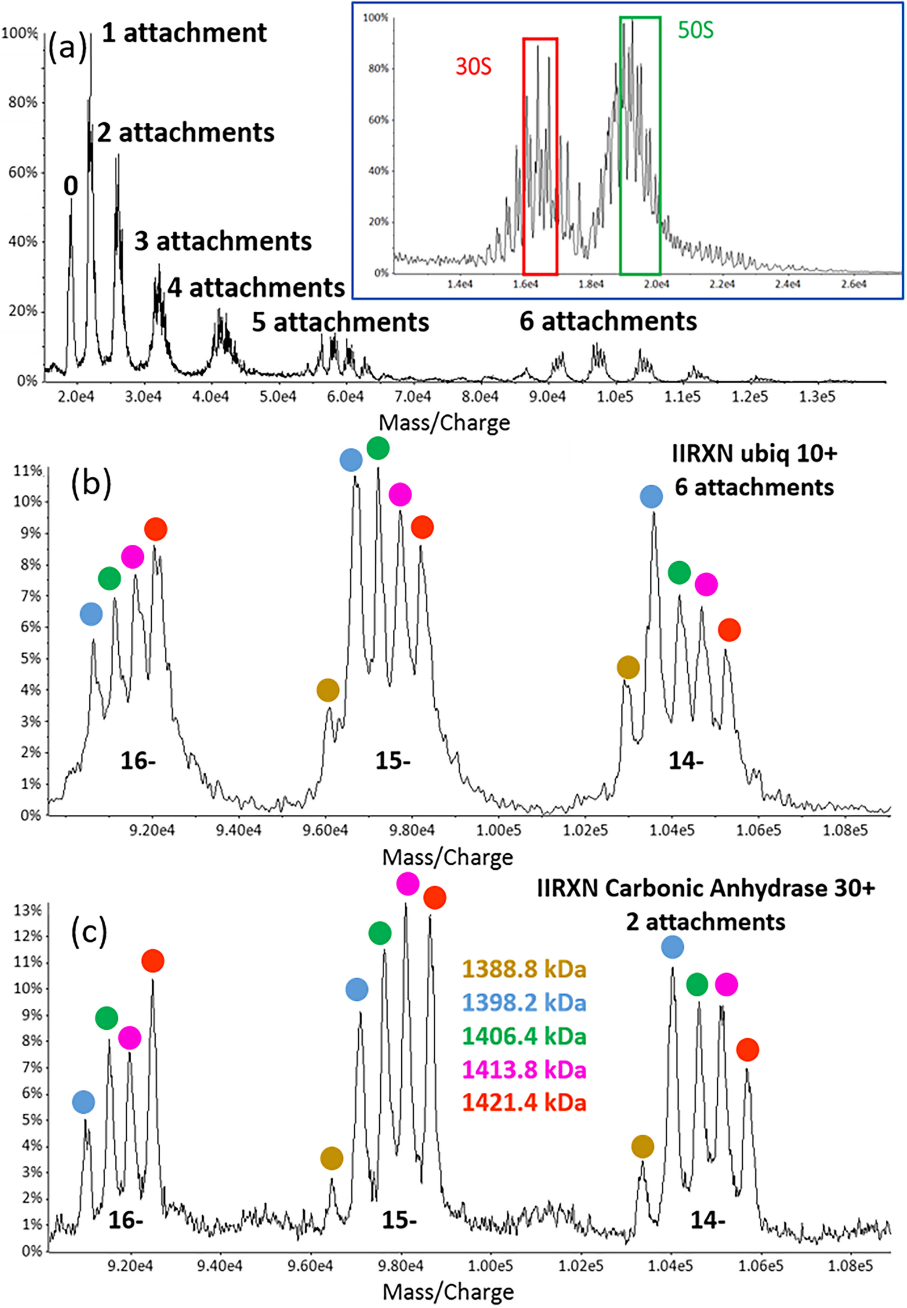
(a) Post‐ion/ion reaction MS after the attachment of up to six 10^+^ ubiquitin ions to selected anions derived from ESI of *E. coli* ribosome 50S particles. The green box in the insert shows the isolated *m/z* region subjected to ion/ion reaction. (b) Expanded *m/z* region for the sixth attachment of ubiquitin 10^+^ reagent ions, which reveals the presence of five major components in the 50S population. (c) Expanded *m/z* region for the second attachment of carbonic anhydrase 30^+^ ions to the same precursor ion population associated with the green box in the insert of (a) and the experiment leading to (b). Equivalent information is present in the spectra of (b) and (c) (see reference 94). Adapted with permission from Reference 94, copyright American Chemical Society.

Two examples of PRIZM experiments applied to a mixture of negatively charged *E. coli* ribosome 50S particles using MIA are summarized in Figure 6 [95]. The insert in Figure 6a shows the negative ESI‐MS of the *E. coli* ribosome under conditions in which ions of the 30S and 50S particles are observed. The green box shows the selected *m/z* window which contains ions of 50S subjected to the MIA experiment. Figure 6a shows the post‐ion/ion reaction spectrum derived from the MIA of up to six 10^+^ ubiquitin cations. Figure 6b shows an expanded view of the *m/z* region with ions generated by the sixth attachment, revealing three charge states for each of five major components within the selected 50S particle ion population. Figure 6c shows the expanded *m/z* region from another MIA experiment in which two 30^+^ cations of carbonic anhydrase are reacted with the same selected ribosome 50S precursor ion population (green box). Both experiments provide reduction by 60 charges and facilitate mass determination for the five major components of the 50S particles. This demonstrates the flexibility associated with a MIA experiment, as multiple reagent ions can be selected to achieve a similar extent of charge reduction.

### 2.2 PRIZM and Multicomponent Mixtures

**FIGURE 7:**
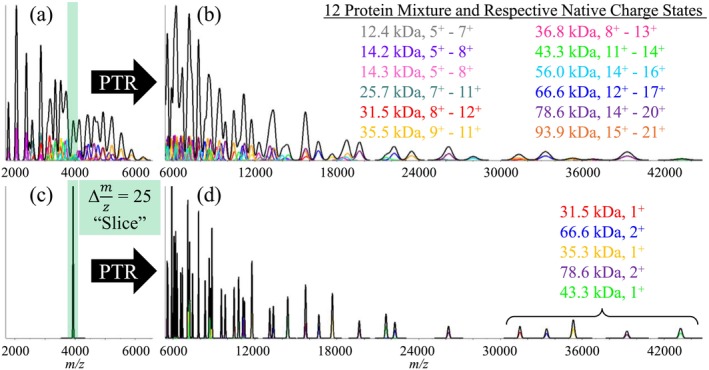
(a) ESI‐MS of a 12‐component mixture ranging in mass from 12.4 to 93.9 kDa. (b) Post‐ion/ion proton transfer mass spectrum of the mixture of ions in (a). (c) Ion isolation of a *m/z* 25 slice from (a). (d) Post‐ion/ion proton transfer mass spectrum of the mixture of ions in (c).

Another scenario in which a mass spectrum may become uninterpretable due to extensive peak overlap arises when there is a highly complex mixture of components. While each component may individually give readily resolved charge state distributions, their combination can result in an unresolved “blob”. Even when individual peaks can be resolved, uncertainty in which peaks are related to one another can lead to errors in assigning masses to mixture components using common approaches. A simulation of a 12‐component mixture, each producing overlapping charge state distributions, illustrates the utility of a PRIZM experiment, as shown in Figure 7. Figure 7a shows the simulated ESI‐MS of the 12‐component mixture of masses and charges shown in the insert in Figure 7b. Figure 7b shows the post‐ion/ion proton transfer reaction spectrum of the full mixture. Figure 7c shows a 25 *m/z* slice of the mixture in Figure 7a, and Figure 7d shows the post‐ion/ion proton transfer spectrum of the 25 *m/z* slice of the original mass spectrum. Note that the peaks in Figure 7d are somewhat narrower than those of Figure 7b as a result of the *m/z* selection and that there are fewer peaks in Figure 7d due to the fact that the slice contains signals from fewer analytes than does the full spectrum of Figure 7a.

**FIGURE 8:**
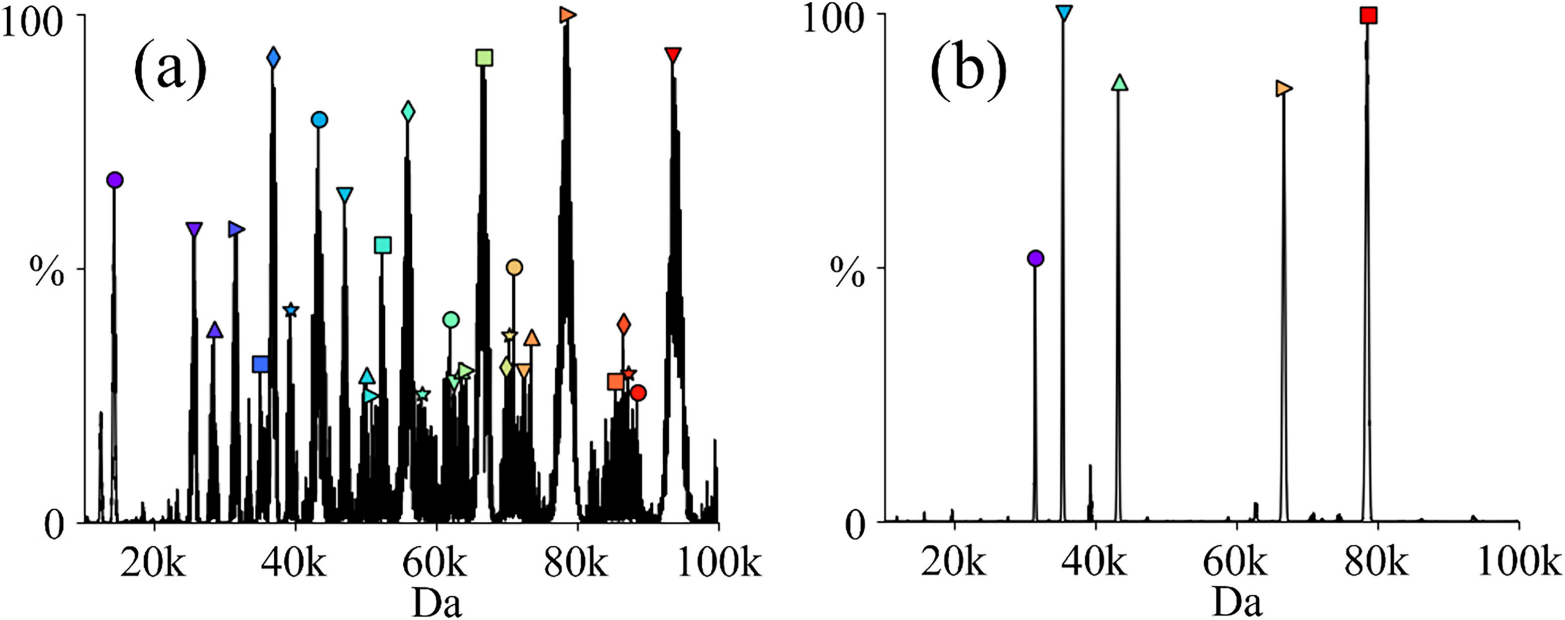
(a) Zero‐charge deconvolution of the ion/ion proton transfer experiment applied to the mixture of ions in Figure 7a (see Figure 7b). (b) Zero‐charge deconvolution of the ion/ion proton transfer experiment applied to the mixture of ions in Figure 7c (see Figure 7d).

Results of zero‐charge deconvolutions by the UniDec [19] program using identical parameters applied to the post‐ion/ion proton transfer data taken to *m/z* 95 000 for the experiments reflected in Figure 7a,b as well as Figure 7c,d are shown in Figure 8a,b, respectively. The zero‐charge mass spectrum of Figure 7b (see Figure 8a) yields more than 30 peaks with relative intensities greater than 25%, most of which are false positives. These errors arise from significant peak overlap in the simulated data. In contrast, the deconvolution of Figure 7d, corresponding to the 25 *m/z* slice, shows five major components, all matching expected masses in the mixture. These figures also illustrate how the width of the selected *m/z* window relates to extent to which charge reduction must be performed in order to minimize false positives. In the absence of ion isolation, the extent of charge reduction was not sufficient to minimize spectral overlap, resulting in a high degree of ambiguity in establishing analyte ion charge states.

**FIGURE 9:**
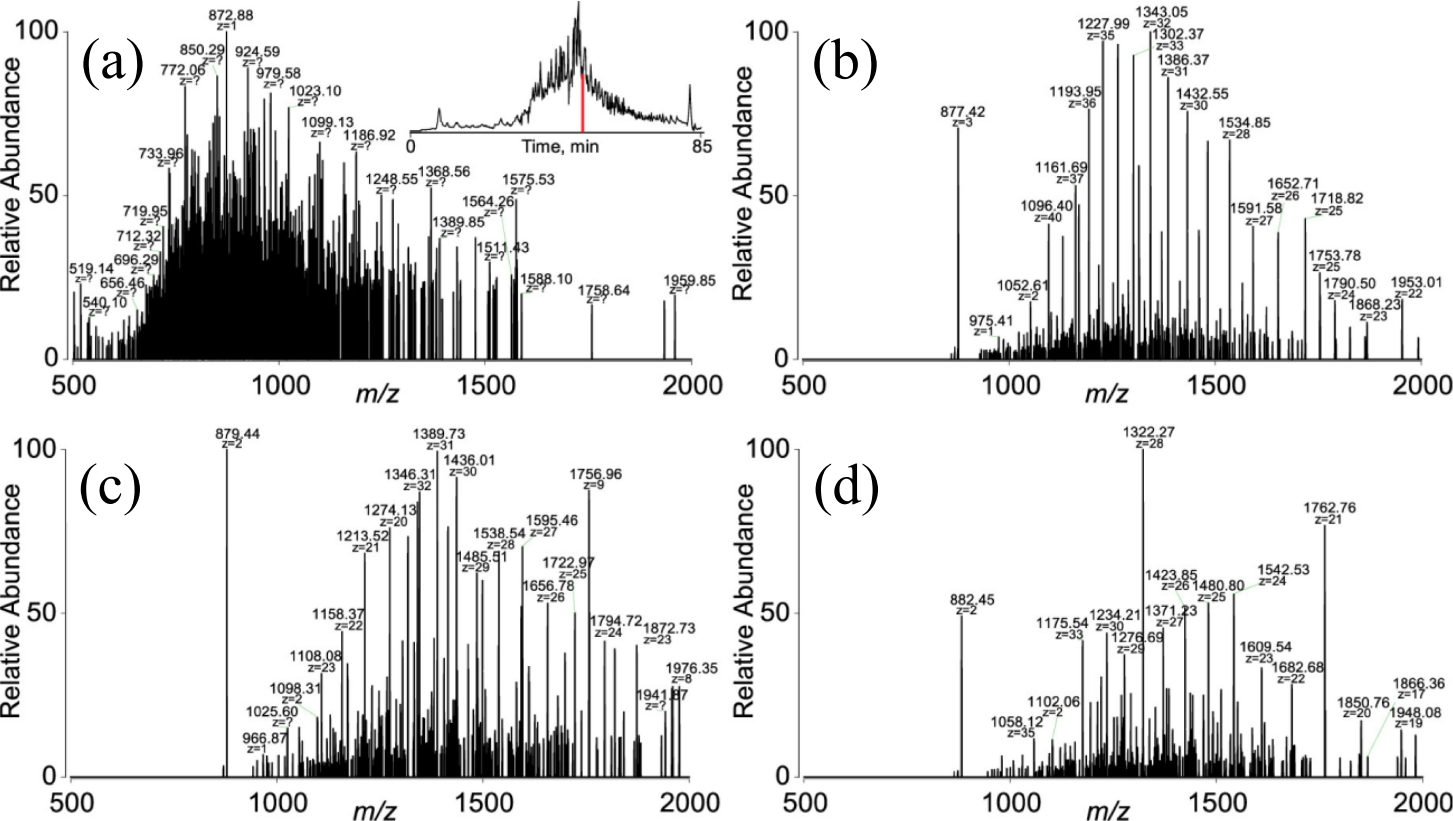
(a) ESI‐MS at 52‐min retention time of a GELFrEE fraction of *P. aeruginosa* . (b) 1.5 *m/z* selection of *m/z* 878 followed by proton transfer charge reduction. (c) 1.5 *m/z* selection of *m/z* 879.5 followed by proton transfer charge reduction. (b) 1.5 *m/z* selection of *m/z* 882.5 followed by proton transfer charge reduction. Adapted with permission from Reference 95, copyright American Chemical Society.

The simulations in Figures 7 and 8 demonstrate the improved confidence with which charges and masses can be assigned by performing a selection step prior to charge reduction (i.e., a PRIZM procedure), even for a relatively simple mixture. Another possible advantage of charge state reduction of mixtures is the improved ability to detect individual components in the mixture that would otherwise be obscured by extensive signal overlap in the initial ESI‐MS. A particularly noteworthy example of the benefit of the PRIZM procedure for top‐down protein mixture analysis was demonstrated in an experiment termed “targeted proton transfer charge reduction” (tPTCR) [96]. In tPTCR, a 1.5 *m/z* window/slice from an ESI‐MS is selected for a short proton transfer ion/ion reaction period (e.g., 8 ms) to generate mass information from the selected *m/z* windows. Figure 9a shows the ESI‐MS of a portion of a chromatographic separation of GELFrEE fraction (see insert), and Figures 9b–d show post‐ion/ion proton transfer spectra for selected regions around *m/z* 878, 879.5, and 882,5, respectively, at the same retention time. According to the report, deconvolution of the data in Figure 9a returned one proteoform mass, whereas the spectra of Figure 9b–d returned four, three, and three unique masses, respectively. The full work‐flow also included a fragmentation step in alternating fashion to enable protein identification. When applied to GELFrEE fractions of *Pseudomonas aeruginosa* ( *P. aeruginosa)* (with proteins 30–60 kDa in mass), the tPTCR approach yielded 192 identifications of UniProt entries (1% false discovery rate) compared to 85 identifications using a previously described medium/high approach for high throughput top‐down proteomics analysis by the same group [97].

**FIGURE 10:**
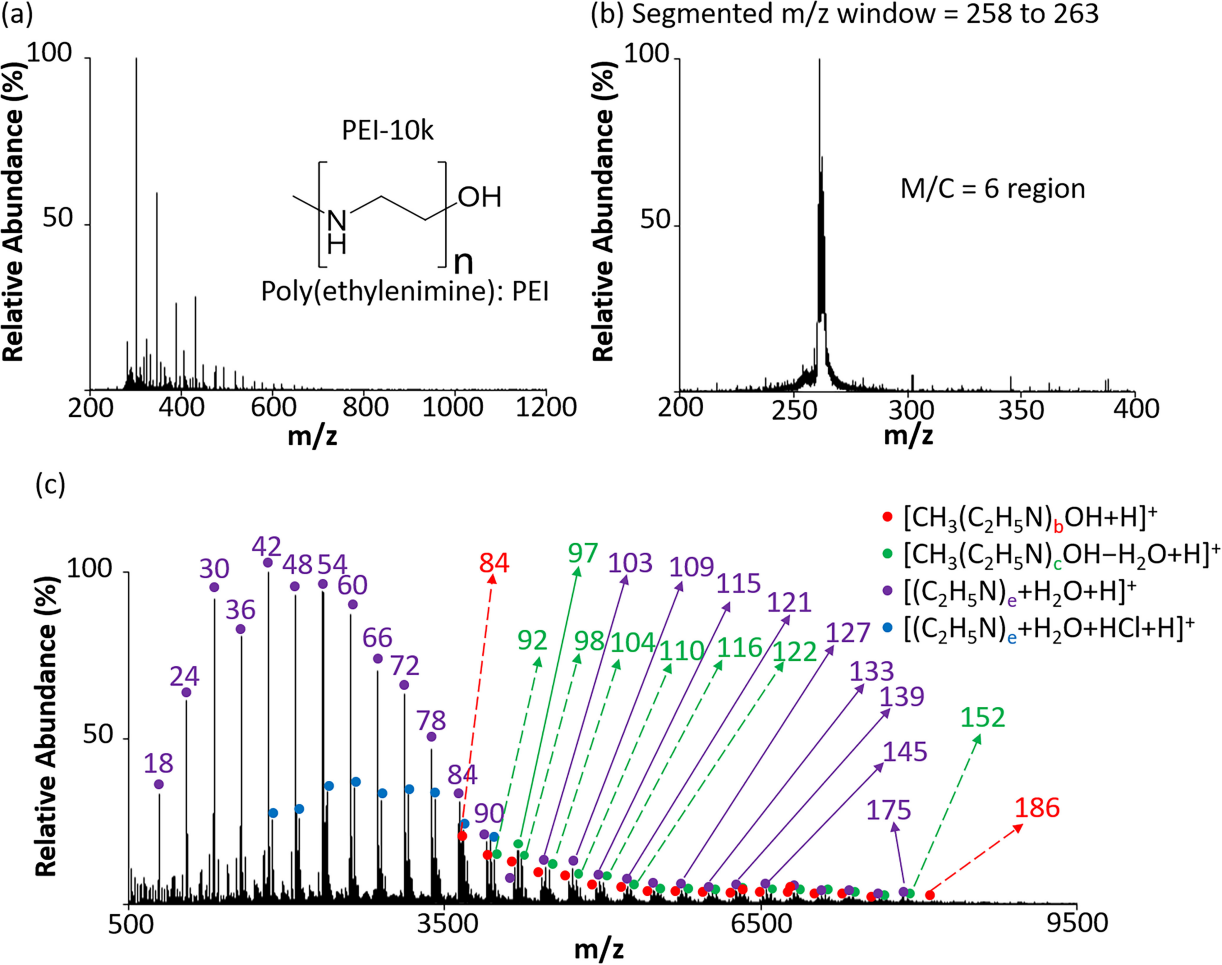
(a) Positive ESI‐MS of poly (ethylenimine). (b) Ion isolation of *m/z* 258–263, which encompasses M/C = 6. (c) Proton transfer charge reduction to the 1^+^ charge state of the mixture of ions isolated in (b). Adapted with permission from Reference 97, copyright American Chemical Society.

A unique case of signal concentration within narrow *m/z* regions can occur while analyzing homopolymers via ESI. Components of polydisperse homopolymers with the same monomer‐to‐charge (M/C) ratio exhibit similar *m/z* values, differing only by the combined mass of their terminal groups. A PRIZM experiment involving the selection of ions with a fixed M/C ratio can serve as an efficient method for characterizing polymer distributions. Figure 10 [98] illustrates this approach using poly (ethylenimine) (PEI), CH_3_(C_2_H_5_N)_n_OH, with a labeled molecular weight of 10 kDa. Figure 10a shows the mass spectrum from directly infusing of PEI‐10k solution to ESI‐MS in positive ion mode. The signal is predominantly concentrated at low *m/z* values, with regions of high relative abundance corresponding to expected M/C values. Figure 10b shows the isolation of ions with the range of *m/z* 258–263, which is expected to contain the ions with M/C = 6 (i.e., one charge for every six monomer units). Figure 10c shows the post‐ion/ion proton transfer spectrum over the region of singly‐charged product ions. Four distributions of ions were found to be present in the isolated *m/z* region, all of which showed a mass spacing of six monomer units, as expected. It was noted that the multiply‐charged PEI ions are particularly fragile, with losses of H_2_O and CH_3_OH being prominent. Also, HCl adducts (the PEI was delivered as the HCl salt) and water adducts were observed. The width of the isolation window was sufficiently wide to encompass such products. Note that CH_3_OH loss effectively removes the end groups so that all of the M/C = 6 ions that lose methanol fall at the same *m/z* value.

## 3 Summary

PRIZM, a type of tandem mass spectrometry experiment, involves the *m/z* selection of one or more precursor ions, charge state reduction, and mass analysis. A PRIZM experiment facilitates charge state determination of multiply‐charged ions when conventional approaches, such as utilizing isotope spacings, spacings between adducts, and spacings between adjacent charge states, are compromised due to extensive spectral overlap. The PRIZM approach can also reveal components in a mixture that differ in mass and charge but have similar *m/z* ratios, as often observed in ESI‐MS. Charge state reduction approaches include ion/molecule proton transfer reactions, ion/ion proton transfer, ion attachment ion/ion reactions, electron transfer ion/ion reactions, and electron capture. PRIZM experiments can be conducted, in principle, on any tandem mass spectrometer platform, provided that a suitable charge reduction approach is available and that the mass analyzers have the upper *m/z* limit to accommodate the charge‐reduced product ions. The number of components that can be separated by charge reduction depends upon the extent of charge reduction as peak separation generally increases as charge states decrease. Therefore, the width of the ion isolation window, which is a determinant in the initial mixture complexity, is inversely related to the extent of charge state reduction and should be carefully considered to achieve a successful PRIZM experiment. Two general applications have emerged for experiments of this type. The first involves systems with one or a few major components that are themselves heterogeneous, as is often encountered in native mass spectrometry. The second focuses on the application of complex mixtures comprised of many components, such as the ESI‐MS of protein mixtures and homopolymers. These examples demonstrate how PRIZM can aid in both the analysis of heterogeneous systems and the deconvolution of complex mixtures. In conclusion, PRIZM is a powerful strategy to obtain accurate charge state and mass information of individual components in complex mixtures undergoing ESI.

## Author Contributions

**Nicolas J. Pizzala:** visualization, writing – original draft preparation. **Hsi‐Chun Chao:** visualization, writing – original draft preparation. **Boukar K.S. Faye:** visualization, writing – original draft preparation **Scott A. McLuckey:** resources, supervision, visualization, writing – review and editing.

## Conflicts of Interest

The authors declare no conflicts of interest.

## Data Availability

Data sharing is not applicable to this article as no datasets were generated or analyzed during the current study.
